# Procalcitonin Detection Using Immunomagnetic Beads-Mediated Surface-Enhanced Raman Spectroscopy

**DOI:** 10.3390/bios14040164

**Published:** 2024-03-29

**Authors:** Jiayue Huang, Dagan Zhang, Yan Zu, Lexiang Zhang

**Affiliations:** 1 Joint Centre of Translational Medicine, the First Affiliated Hospital of Wenzhou Medical University, Wenzhou 325035, China; hjy54321@163.com; 2 Oujiang Laboratory (Zhejiang Lab for Regenerative Medicine, Vision and Brain Health), Wenzhou Institute, University of Chinese Academy of Sciences, Wenzhou 325000, China; 3 State Key Laboratory of Targeting Oncology, National Center for International Research of Bio-targeting Theranostics, Guangxi Key Laboratory of Bio-targeting Theranostics, Collaborative In-novation Center for Targeting Tumor Diagnosis and Therapy, Guangxi Medical University, Nanning 530021, China; 4 Department of Rheumatology and Immunology, Nanjing Drum Tower Hospital, School of Biological Science and Medical Engineering, Southeast University, Nanjing 210096, China

**Keywords:** SERS, PCT, magnetic nanoparticles, gold nanoparticles, Raman probe

## Abstract

The early detection of procalcitonin (PCT) is crucial for diagnosing bacterial infections due to its high sensitivity and specificity. While colloidal gold colorimetric and immune-chemiluminescence methods are commonly employed in clinical detection, the former lacks sensitivity, and the latter faces challenges with a brief luminescence process and an elevated background. Here, we introduce a novel approach for the quantitative analysis of PCT using surface-enhanced Raman spectroscopy (SERS), leveraging the enhanced properties of metal nanoparticles. Simultaneously, we employed a magnetic nanoparticle coating and surface biofunctionalization modification to immobilize PCT-trapping antibodies, creating the required immune substrates. The resulting magnetic nanoparticles and antibody complexes, acting as carriers and recognition units, exhibited superparamagnetism and the specific recognition of biomarkers. Then, this complex efficiently underwent magnetic separation with an applied magnetic field, streamlining the cumbersome steps of traditional ELISA and significantly reducing the detection time. In conclusion, the exploration of immunomagnetic bead detection technology based on surface-enhanced Raman spectroscopy holds crucial practical significance for the sensitive detection of PCT.

## 1. Introduction

Sepsis, a severe condition stemming from an aberrant immune response to infection, poses a substantial threat by inducing organ dysfunction, potentially leading to septic shock and death [1]. Timely intervention significantly improves patient survival; however, the existing detection methods definitely face limitations. Blood cultures are time-intensive, nucleic acid testing demands specialized equipment and skilled personnel, and protein testing lacks specificity. Procalcitonin (PCT), a protein marker exhibiting high sensitivity and specificity, has emerged as a valuable diagnostic tool for severe bacterial infections and sepsis, guiding antibiotic usage effectively. To harness the potential of PCT detection, we propose utilizing surface-enhanced Raman spectroscopy (SERS), a surface-sensitive technique employing nanostructures to amplify Raman scattering and identify molecular fingerprint features [2]. SERS significantly enhances the Raman signal of molecules adsorbed on nanostructures by leveraging the plasmon resonance effect of metals like Ag and Au, achieving ultra-high detection sensitivity. Noteworthy advantages of SERS include its fast speed, non-destructiveness, water compatibility, and suitability for real-time protein conformation detection at physiological concentrations [3,4,5]. Consequently, SERS presents a promising avenue for PCT detection. In light of the urgent need for a timely and sensitive PCT detection method, our study aims to explore the potential of SERS combined with immunomagnetic beads.

SERS and immunomagnetic beads have revolutionized biological detection technologies, providing an efficient, specific, and sensitive system for clinical detection [6,7,8,9]. The detection conditions of this system are mild, and it is widely used in a variety of interfaces. This approach exhibits versatility across various interfaces, particularly in liquid-phase systems. The advent of handheld Raman spectrometers has further democratized SERS application, eliminating the high costs and complexity associated with larger instruments. Raman reporter molecules and detection antibodies attach to the surface of gold nanoparticles [10]. After specific binding to the antigen to be tested, the abnormal surface optical phenomenon is significantly enhanced under 633 nm laser irradiation so as to achieve the detection signal’s amplification [11]. A large number of studies have demonstrated the feasibility of this technology [12,13,14]. The emergence of SERS spectrum analysis technology overcomes the shortcomings of chemiluminescence detection technology, such as a short luminescence process and poor reagent stability. However, its clinical application value still needs further exploration. Magnetic nanoparticles (MNPs) are a kind of nanomaterials with superparamagnetic properties, good biocompatibility, and chemical stability. In recent years, they have been widely used in the field of biotechnology, such as in nuclear magnetic resonance technology [3,15,16], targeted drug delivery [17,18,19], cell isolation [20,21], and the labeling and detection of biomarkers associated with various diseases [22,23,24,25]. Among these, Fe_3_O_4_ MNPs have advantages in many nanomaterials due to their small size, high surface area, dispersion stability, easy modification, and magnetic separation. Coating Fe_3_O_4_ nanoparticles with SiO_2_ to form a core–shell structure enhances their stability and facilitates further modifications, including amino and carboxyl group additions. This adaptability allows for the coupling with proteins and nucleotides via chemical covalent bonds, paving the way for biomedical applications [26,27,28].

**Figure 1:**
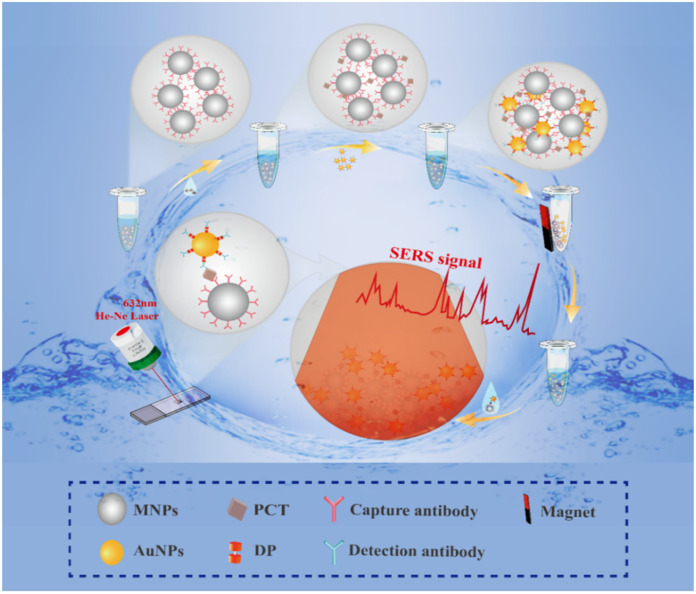
SERS immunoprobe detection system based on magnetic separation.

Here, we explore in depth a cutting-edge technology that cleverly combines magnetic nanoparticle antibody probes with SERS signal amplification technology, providing a new perspective for the detection of target antigen PCT. The core of this technology lies in the structural design shown in Figure 1. First, we adopted a hydrothermal method to synthesize the MNPs. In order to improve the biocompatibility of the MNPs, we put a layer of silica “coat” on them. This not only enhances the stability of MNPs in biological environments but also effectively reduces nonspecific binding and improves their detection accuracy. This structure ensures the efficient coupling of detection antibodies and lays a solid foundation for the subsequent detection process. Subsequently, we enabled the MNPs to be cross-linked with PCT monoclonal antibodies through a series of chemical modifications, such as amination and carboxylation. The successful implementation of this step enabled our probe to accurately capture the target antigen and prepare for the subsequent signal amplification. Next, we attached another monoclonal antibody and the Raman reporter pyridine to the Au nanoparticles (AuNPs), targeting another epitope of PCT. In this way, when the antigen is captured by the MNPs, these AuNPs can combine with it to form an efficient SERS hotspot [29]. Finally, when the excitation light strikes these SERS hot spots, the Raman reporter molecules induce the generation of high-intensity Raman scattering spectra. This spectral signal not only has an extremely high sensitivity but can also reflect the presence or absence of the antigen, thereby enabling the trace detection of the PCT antigen. The innovation of this technology is that it combines the specificity of the antigen–antibody reaction with the ultra-sensitivity of SERS, allowing one to accurately detect the PCT antigen at extremely low concentrations. In addition, using MNPs as a reaction carrier also allows one to use magnetic separation technology to quickly separate the target antigen from other components in the test sample, further improving the efficiency and accuracy of the test. Furthermore, this technology is expected to be combined with portable Raman spectrometers to provide immediate, accurate results for emergency and point-of-care testing [30]. Whether in medical institutions or remote areas, this rapid and sensitive detection method will provide strong support for the early detection and treatment of diseases.

## 2. Results and Discussion

**Figure 2:**
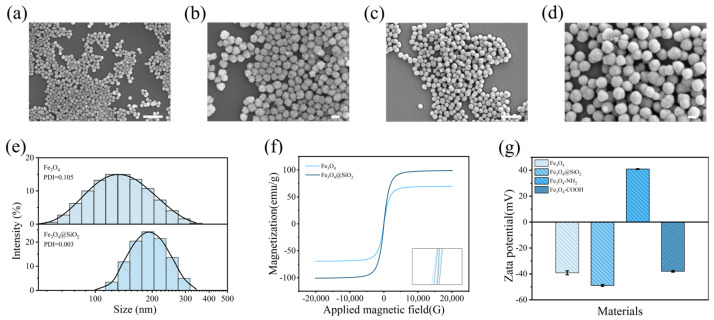
(**a**,**b**) SEM images of Fe_3_O_4_ NPs with scales of 500 nm and 100 nm, respectively; (**c**,**d**) SEM images of Fe_3_O_4_@SiO_2_ NPs with scales of 500 nm and 100 nm, respectively; (**e**) particle size distribution plots of Fe_3_O_4_ NPs and Fe_3_O_4_@SiO_2_ NPs; (**f**) superparamagnetic curves of MNPs; (**g**) zeta potential value changes along MNPs modifications.

In a typical experiment, Fe_3_O_4_ NPs were successfully synthesized through a hydrothermal reaction, and SEM imaging demonstrated their uniformity, dispersion, purity, high yield, and controllable morphology, with a diameter of 100 nm (Figure 2a,b). This reaction allowed for the precise control of NP size by adjusting the water content and other factors, while ethylene glycol was chosen as the solvent to reduce the agent and the protective agent due to its capability in modifying morphology and controlling nucleation. To impart a good stability, biocompatibility, and an easy modification capability to the NPs’ surface, a thin layer of SiO_2_ was sequentially coated using a modified Stober method [31]. The thickness of the silica shell depended on the introduction of ethyl orthosilicate (TEOS). The SEM characterization is shown in Figure 2c,d, and its size and distribution were characterized using a nanoparticle-size meter (Figure 2e). These monodisperse magnetic nanoparticles with a silica shell thickness of approximately 50 nm facilitated signal quantification and prevented NP agglomeration. Despite a one-third reduction in the saturation magnetization values from 70 emu·g^−1^ for the bare NPs, the silicon-coated NPs still maintained a high magnetic responsiveness for instant magnetic separation from the solution (Figure 2f).

By subjecting them to an external magnetic field, MNPs in an aqueous solution rapidly underwent adsorption and aggregation. These NPs exhibited exceptional magnetic responsiveness without hysteresis and could swiftly redisperse in the solution after the magnetic field had been removed (Figure S1). Consequently, following suitable modification, they served as efficient carriers for substance separation. The magnetic separation method offers several advantages over alternative techniques. Primarily, magnetic nanoparticles are cost-effective, and their synthesis process is well-established, enabling straightforward and economical mass production. Furthermore, their versatile application is another key advantage, owing to their compatibility with various modifications. This versatility allows for the easy immobilization of biomolecules such as enzymes, proteins, DNA, or cells onto particles for biomedical applications.

**Figure 3:**
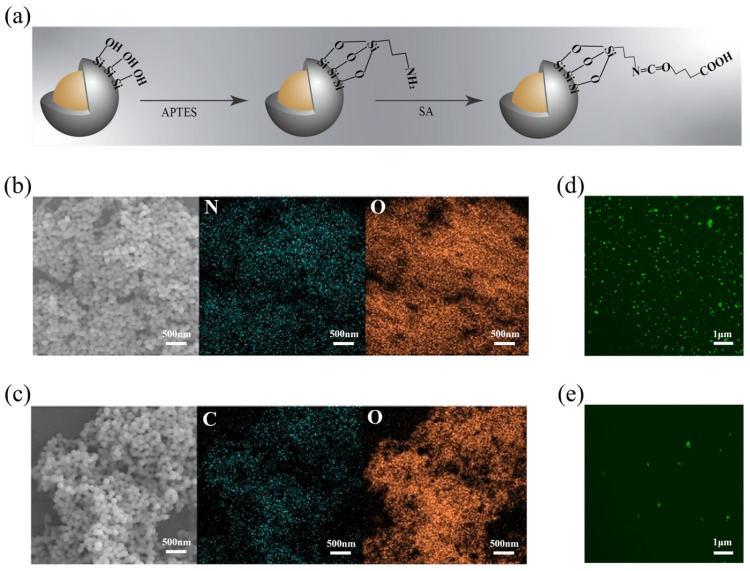
(**a**) Schematic diagram of Fe_3_O_4_@SiO_2_ MNPs surface group modification; (**b**) element analysis by energy spectrum scanning after amination of Fe_3_O_4_@SiO_2_ NPS; (**c**) energy spectrum scanning element analysis of Fe_3_O_4_@SiO_2_ NPS after carboxylated modification; (**d**) fluorescent secondary antibodies were attached to MNPs-immobilized capturing antibodies; and (**e**) fluorescent secondary antibodies were attached to unmodified MNPs.

**Figure 4:**
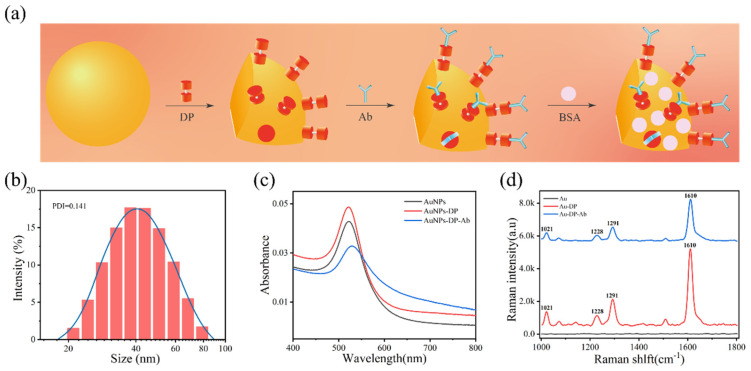
(**a**) Schematic diagram of SERS probe preparation; (**b**) particle size distribution of AuNPs; (**c**) UV absorption spectra of AuNPs and AuNPs after linking pyridine and antibodies; and (**d**) Raman signaling after AuNPs link pyridine and antibodies.

For covalently bonding the captured antibody onto the NP carriers, an accessible surface modification approach was adopted (Figure 3a). Specifically, amino groups were introduced via an APTES source, followed by carboxylation with a succinic anhydride solution. As the immobilization progressed towards antibody linkage, the zeta potential of the NP-dispersed solution decreased to −55 mV in the presence of silica, shifted to 38 mV with the addition of sufficient amine groups, and then dropped to around −35 mV due to the presence of -COOH on the surface (Figure 2g). From the surface element map analysis of Fe_3_O_4_@SiO_2_@NH_2_ and Fe_3_O_4_@SiO_2_@COOH (Figure 3b,c), it can be seen that, after the surface of the magnetic nanoparticles was modified with groups, the signals contributed by the N and C elements were very dense, so it could be proved that, in the amino and carboxyl groups, the amount of grafting is sufficient for subsequent antibody immobilization. Fourier infrared spectroscopy showed the stretching vibration of some groups, and the vibration of the silicon–oxygen bond led to the absorption of infrared light, forming a peak at 1081 cm^−1^, indicating that SiO_2_ was successfully wrapped on the surface of Fe_3_O_4_. However, due to the limited amount of surface modification, the amino and carboxyl group modifications did not show obvious absorption peaks (Figure S2). Subsequently, a 1-Ethyl-3-(3′-dimethylaminopropyl) carbodiimide and N-Hydroxysuccinimide (EDC/NHS) cross-linking scheme was employed to add PCT capture antibodies onto the NPs. This conjugation was well characterized by the successful immobilization of fluorescent-labeled secondary antibodies on the surface (Figure 3d). As a comparison, the control sample with unmodified functional groups revealed only a small amount of nonspecifically adsorbed fluorescent antibodies on the NPs after the same magnetic separation process (Figure 3e). This confirmed that the binding between the NPs and the target antibody was predominantly covalent rather than nonspecific. In addition, the conjugation ratio of antibodies on the NPs was optimized using a BCA protein concentration assay, indicating that 15–25 μg of antibodies could be conjugated per mg of MNPs (Figure S3). To achieve highly sensitive and specific antigen detection, immune SERS probes were formed by absorbing the detection antibodies and the Raman reporter molecules onto the surface of AuNPs (Figure 4a). AuNPs used as tracers for signal amplification were synthesized via the seed growth method [32].

The Raman signal of the reporter molecule labeled on the AuNPs increased cumulatively with the increase in the AuNPs’ particle size. The Raman signal of the labeled reporter molecules on the AuNPs accumulated with the increase in the AuNPs’ particle size, and a SERS probe with a good signal was required in this experiment, but, at the same time, the dispersion, uniformity, and stability of the nanoparticles themselves were good so as to ensure that the labeled molecules and antibodies would not accumulate after they had been connected. The experimental results revealed that freshly prepared gold sol with 50 nm particles achieved an ideal SERS signal pattern, maintaining good dispersion, uniformity, and stability to prevent the agglomeration of the labeled molecules and antibodies before and after conjugation (Figure 4b). In detail, the concentration of sodium citrate and ascorbic acid in the recipe determined the AuNPs’ size, as characterized by the photograph and the SEM image (Figure S4). 4,4-Bipyridine (DP) was selected as the Raman reporter due to its fast labeling and non-toxic properties. Since the antibody is already adsorbed on the DP of AuNPs, only the content of labeled DP needs to be further optimized. (Figure S5). The experimental results indicated that too-little DP led to a weak signal while an excessive amount caused the agglomeration of gold colloids, reducing the signal’s intensity. The optimal labeled amount was determined to be 30 μL.

The absorption spectra of the AuNPs, DP-labeled AuNPs, and immune-SERS probes were detected by ultraviolet-visible near-infrared spectroscopy (UV) under optimal conditions. A red shift in the peak value of 520 nm was clearly observed, demonstrating successful antibody attachment to the DP (Figure 4c). To illustrate the significance of SERS signal amplification, laser microscopy confocal Raman spectrometry was employed to characterize the immune connection and responsive changes in the spectra. It is well documented that the observed Raman signals of labeled Au NPs were significantly enhanced, while they were not affected by later antibody adsorption (Figure 4d). These features paint a promising picture for the high-sensitivity detection of the antigens of interest.

A complete route with a one-step liquid-phase detection condition was developed. This involved capturing antibodies coupled with MNPs and detecting antibodies labeled with gold NPs in PBS. Upon capturing the targeted antibody, a sandwich structure with the antigen was assembled, generating detectable signals. The magnetic core of the assembly facilitated instant magnetic separation, detaching unreacted residuals. Finally, 2.5 μL of the sample aliquot, dropped onto the silicon chip, underwent Raman characterization corresponding to the 1612 cm^−1^ bands from the reference molecules, enabling the quantification of its concentration in an accessible and efficient manner.

**Figure 5:**
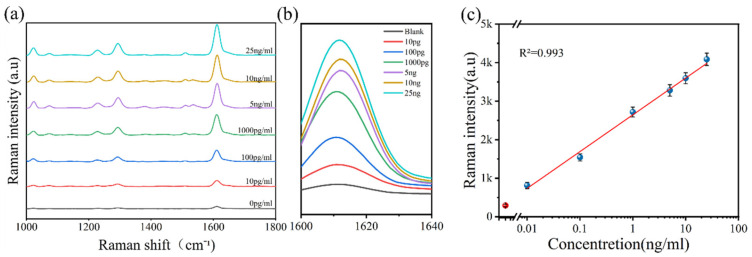
(**a**,**b**) Mean of SERS spectra of six parallel testing results of PCT antigen at different concentrations; and (**c**) correlation curves of PCT concentration and Raman signal intensity.

**Figure 6:**
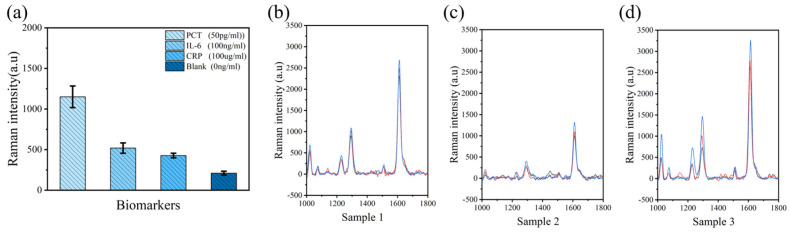
(**a**) Specificity of the SERS immunodetection system; (**b**) Raman spectra collected at three random points of sample 1; (**c**) Raman spectra collected at three random points of sample 2; and (**d**) Raman spectra collected at three random points of sample 3.

Experiments with PCT antigens covering a wide concentration range were conducted under optimized conditions to construct standard curves (Figure 5c). As expected, our method exhibited a linear response relationship along PCT concentrations from 10 pg/mL to 25 ng/mL. The minimum detection limit was defined as the mean plus three times the standard deviation of 20 replicates of blank samples. Of note, the minimum detection limit of PCT was approximately 5.3 pg/mL through the blank sample, while the clinically set cutoff value of PCT is often 50 pg/mL (Figure S6). To evaluate the selectivity of the method, the impact of other markers present in human serum on PCT detection was investigated. Using PCT with a clinical cutoff mass concentration of 50 pg/mL as the benchmark, high concentrations of IL-6 (100 ng/mL) and CRP (100 μg/mL) in each index series of standard solutions were employed as interferences to verify the method’s specificity. The same reaction steps were incubated, and the peak at 1612 cm^−1^ was quantitatively analyzed by Raman spectroscopy. The results in Figure 6a show that other biomarkers with concentrations much higher than PCT only produced weak Raman response values comparable to the blank control and, therefore, did not cause significant interference to the PCT detection system. In contrast, only 50 pg/mL PCT produced a strong response signal, confirming the good specificity of the proposed detection method. To further verify the practical potential of this method, three clinical serum samples with PCT concentrations ranging from 0.024 to 1.500 mg/mL were measured and compared with the detection results of the chemiluminescence instruments used in clinical laboratories (Figure 6b–d and Table 1). Gratifyingly, both methods yielded consistent outcomes with no significant differences, as indicated by the paired t-test (*p* > 0.05).

## 3. Conclusions

In summary, a novel method for the rapid and sensitive detection of procalcitonin (PCT) using surface-enhanced Raman spectroscopy (SERS) technology and immunomagnetic beads has been introduced. Conventional PCT detection methods mainly include semi-quantitative methods and quantitative methods, such as chemiluminescence, colloidal gold immunochromatography, radioimmunoassay, etc. These methods are primarily used for the diagnosis and monitoring of systemic inflammation caused by bacterial infections. Although these methods are widely used in clinical medicine, they have some significant problems, such as a high chemiluminescence background and a short luminescence process, colloidal gold immunochromatography is not quantitative, and a radioimmunoassay uses harmful radioactive elements. In contrast, SERS methods may have a higher sensitivity and a faster detection speed and require minimal sample volumes and lower costs, making them a better choice in some cases.

With the widespread application of portable Raman spectrometers in recent years, we have reason to believe that this method can not only meet the needs of on-site detection but also further reduce the detection costs. Among the existing clinical PCT detection methods, colloidal gold immunochromatography is the method with the lowest cost. Its detection cost is between 1.2 CNY/part and 1.8 CNY/part. However, the cost of the SERS detection method we developed will be lower than 0.8 CNY per use. Our developed detection system can achieve single-molecule detection of PCT, with the characteristics of a trace sample quantitative analysis, and does not require a complex sample pretreatment, just requiring one to drop the sample onto the silicon chip to obtain the spectral signal. The system is not limited to PCT detection but also provides a valuable technical reference for detecting other biomarkers. This detection method is simple, and the detection results are reliable. The method uses a combination of SERS technology and immunomagnetic beads, which improves the intensity and specificity of the detection signal and avoids the interference of other factors. In summary, this detection method has broad application prospects for the detection of various biomarkers in clinical practice and provides a fast and sensitive new detection scheme for disease diagnosis.

## 4. Experimental Section

**Materials.** Ethylene glycol (EG), anhydrous ferric chloride (FeCl_3_, 99.9%), sodium acetate (NaAc), poly (4-styrene sulfonic acid-maleic acid) sodium salt (PSSMA, Mw ≈ 20,000, SS:MA = 1:1), N, n-dimethylformamide (DMF), toluene, 3-Aminopropyltriethoxysilane (APTES), Succinic anhydride, 1- (3-Dimethylaminopropyl)-3-ethylcarbodiimide hydrochloride (EDC), N-Hydroxysulfosuccinimide (NHS), and bovine serum albumin (BSA) protein were purchased from Aladin (Shanghai, China). Sodium hydroxide (NaOH), tetraethoxysilane (TEOS), ammonium hydroxide (NH_3_·H_2_O, 25.0~28.0%), L-ascorbic acid (Vitamin C,AA), dimethyl sulfoxide (DMSO), 4,4-Bipyridine, and 4-morpho-ethanesulfonic acid (MES) were sourced from Shanghai Maclin Biochemical Co. (Shanghai, China) Ethanol, gold chloride (HAuCl_4_), and trisodium citrate were taken from Sinopharm Chemical Reagent Co., Ltd. (Shanghai, China). The PCT monoclonal antibody, PCT antigen, IL-6 antigen, and CRP antigen were purchased from Nanjing Okai Biotechnology Co., Ltd. (Nanjing, China). The phosphate buffer (1x, 10 mM, pH 7.4) and borate buffer (2 mM, pH8.2) were sourced from Voredas experimental reagent supplies. EG was pre-dried with a molecular sieve (type 3A), while the other chemicals were used according to the received standard. The water used in all the experiments was sourced from a Milliq 185 water purification system (Millipore, Bedford, MA, USA), with a resistivity higher than 18 MΩ·cm, and was used directly without further purification.

**Preparation of MNPs.** Firstly, 15 mL EG, 0.26 g FeCI_3_, 1.2 g NaAc, 0.4 g PSSMA, 4.5 mg AA, and 20 μL deionized water were successively added to a 20 mL glass bottle. After ultrasonic stirring for 30 min and after stirring with a magnetic force for 5 h, 0.24 g NAOH was added, and, after stirring for 5 h, the mixture was placed in a Muffle furnace at 190 °C for 10 h. After cooling to room temperature, the mixture was removed, washed with ethanol and deionization water for three times each, and, finally, dispersed in 6 mL deionized water and stored at 4 °C for later use.

**Preparation of MNPs Antibody.** A total of 3 mL was removed from the above MNPs’ aqueous solution and 3 mL of deionized water, 40 mL ethanol, and 2 mL ammonia were added in turn. The mixed solution was ultrasonically stirred for 20 min, then placed in a three-orifice flask and stirred with a stirring paddle for 15 min. A total of 300 μL TEOS was added at a rate of 20 s per drop and then continuously stirred for 1 h, washed with deionized water and ethanol for three times, and, finally, dispersed in 6 mL ethanol to obtain Fe_3_O_4_@SiO_2_. Then, 2 mL of the above Fe_3_O_4_@SiO_2_ was taken and washed with ethanol and DMF; then, 20 mL DMF and 8 mL toluene were added in turn, ultrasonically stirred for 5 min, put in a 100 mL flask, and stirred at 70 °C for 8 h, at which point, the heating was stopped and the product was stirred at room temperature for 2 h, left at room temperature overnight, cleaned with ethanol, and dissolved in a DMSO solution containing 1% butyric anhydride. It was then shocked overnight. Finally, the carboxyl group was activated using an MES buffer containing EDC/NHS and then conjugated with the trapping antibody (Ab_1_), which was blocked by BSA. The magnetic nanoparticle antibody substrate was prepared and stored at 4 °C for future use.

**Preparation of AuNPs.** A total of 50 mL of HAuCL_4_ solution (0.29 mmol/L) was heated until boiling and kept for 15 min; then, 1 mL 1% sodium citrate was rapidly added and kept boiling for 20 min, until the solution turned wine-red. Finally, the solution was cooled to room temperature under uniform stirring to obtain gold sol seeds. Then, 10 mL of gold sol seeds were added into 25 mL of rapidly stirred deionized water, 0.5 mL HAuCl_4_ solution (29 mmol/L) was quickly added, and, finally, 0.5 mL of ascorbic acid (0.1 mol/L) was added, and the stirring continued for 30 min to obtain gold sol.

**Preparation of SERS Antibody.** First, 1 mL of the gold sol prepared above was added to 30 μL DP (0.01 mM), shook at room temperature for 5–10 min, and centrifuged at 3000 rpm for 25 min. The supernatant was discarded, and the precipitation was redissolved in BBS, and the detection antibody (Ab_2_) was added, incubated for 2 h, and then centrifuged at 3000 rpm for 25 min. After the supernatant had been blocked with BSA for 1 h and centrifuged again at 3000 rpm for 25 min, the supernatant was redissolved in PBS containing BSA (0.01%). SERS probes were prepared and stored at 4 degrees for further use.

**Preparation of matrix solution and PCT series standard solution.** PBS buffer containing BSA (3%) was configured as the matrix solution, and the PCT antigen was diluted with the matrix solution to concentrations of 0, 10, 100, 1000, 5000, 10,000, and 25,000 pg/mL.

**Magnetic immunoanalysis based on SERS.** First, 50 μL MNPs-Ab was added into the PCT standard solution to be tested and incubated at 37 °C for 30 min. PBS containing 0.05% Tween 20 (PBST) was used as the washing buffer. After washing the compound three times, 50 μL SERS probes were added and incubated at 37 °C for 30 min, and PBST was used again three times. Finally, it was reconstituted with 20 μL PBS and added dropwise onto a silicon chip. The He-Ne laser used had a wavelength of 633 nm, and a 50 objective with a long working distance collected the Raman scattering signal. The laser power to excite the sample was 25 mW, and the integration time of each SERS spectrum was 10 s. Three points were randomly moved on the silicon wafer to detect the spectral region of 1000–1800 cm ^−1^. The peak Raman intensity was measured at the average intensity at 1612 cm^−1^. When testing clinical serum samples, take 50 μL of serum and use the same detection steps as above.

**Chemiluminescence-based immunoassay.** Add 2 mL of PCT serum sample to the chemiluminescence kit including antibodies, markers, and buffers. Through electromagnetic action, the magnetic beads are adsorbed on the electrode surface to remove substances that are not bound to the magnetic beads. Applying a certain voltage energy to the electrode causes the complex to chemiluminescence. Luminous intensity is measured with a photomultiplier. The Elecsys 2010 software automatically calculates the test results.

**Characterization.** Field emission scanning electron microscopy (Hitachi, SU8010); UV-Vis-NIR spectroscopy (Agilent, CARY 5000); confocal Raman microscopy (Renishaw, inViaQontor); particle size analyzer (Malvern, ZEN3600); and Fourier transform infrared spectroscopy (Brooke, TensorII).

## Figures and Tables

**Table 1:** Comparison between SERS detection and chemiluminescence method.

Sample (no.)	Chemiluminescence (ng/mL)	SERS (ng/mL)	Recovery (%)
1	0.616	0.678	90.9
2	0.024	0.018	85.7
3	1.500	1.415	106.0

## Data Availability

Data available within Supplementary Materials or on request from the authors.

## Supplementary Materials

The following supporting information can be downloaded at: https://www.mdpi.com/article/10.3390/bios14040164/s1, Supplementary Materials accompanying this paper are available.
