# Supplementary material for: Procalcitonin Detection Using Immunomagnetic Beads-Mediated Surface-Enhanced Raman Spectroscopy
Source: Biosensors (Basel). 2024 Mar 29;14(4):164. doi: 10.3390/bios14040164 (PMC11048292; doi:10.3390/bios14040164)
Supplement: Supplementary file 1 [file biosensors-14-00164-s001.zip › biosensors-2911202-supplementary.pdf]

biosensors

Supplementary Materials

Procalcitonin Detection Using Immunomagnetic Beads-Mediated Surface-Enhanced Raman Spectroscopy

Jiayue Huang [1,2,3], Dagan Zhang [4,*], Yan Zu [2,*] and Lexiang Zhang [1,2,*]

1 Joint Centre of Translational Medicine, the First Affiliated Hospital of Wenzhou Medical University, Wenzhou 325035, China; hjy54321@163.com
2 Oujiang Laboratory (Zhejiang Lab for Regenerative Medicine, Vision and Brain Health), Wenzhou Institute, University of Chinese Academy of Sciences, Wenzhou 325000, China
3 State Key Laboratory of Targeting Oncology, National Center for International Research of Bio-targeting Theranostics, Guangxi Key Laboratory of Bio-targeting Theranostics, Collaborative In-novation Center for Targeting Tumor Diagnosis and Therapy, Guangxi Medical University, Nanning 530021, China
4 Department of Rheumatology and Immunology, Nanjing Drum Tower Hospital, School of Biological Science and Medical Engineering, Southeast University, Nanjing 210096, China
* Correspondence: dgzhang@njglyy.com (D.Z.); zuyan@ucas.ac.cn (Y.Z.); zhanglx@ucas.edu.cn (L.Z.)

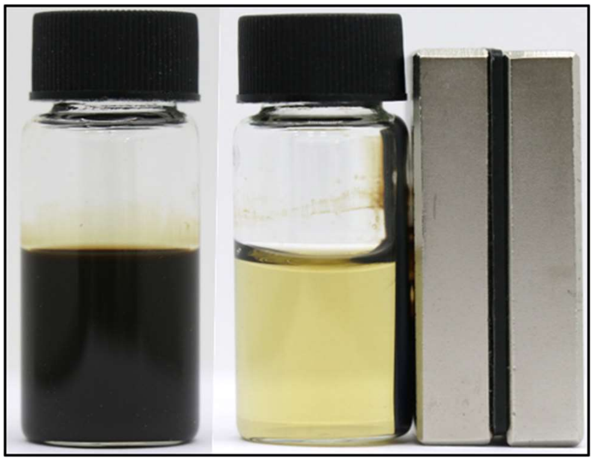

Figure S1. Magnetic adsorption diagram of magnetic nanoparticles in an aqueous solution.

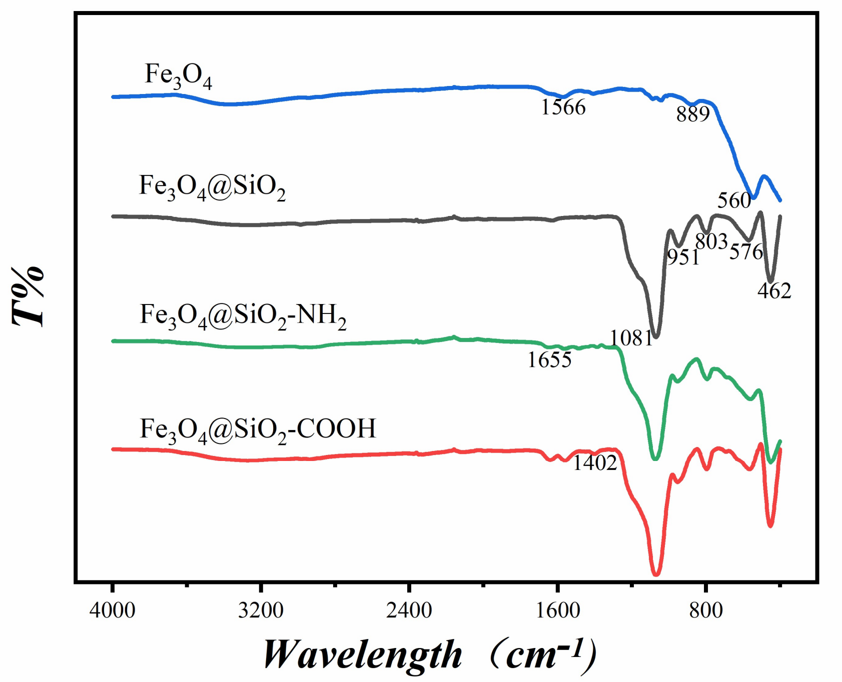

Figure S2. Infrared spectra of Fe_3O_4, $Fe_3O_4@SiO_2$, $Fe_3O_4@SiO_2$-NH_2, and $Fe_3O_4@SiO_2$-COOH.

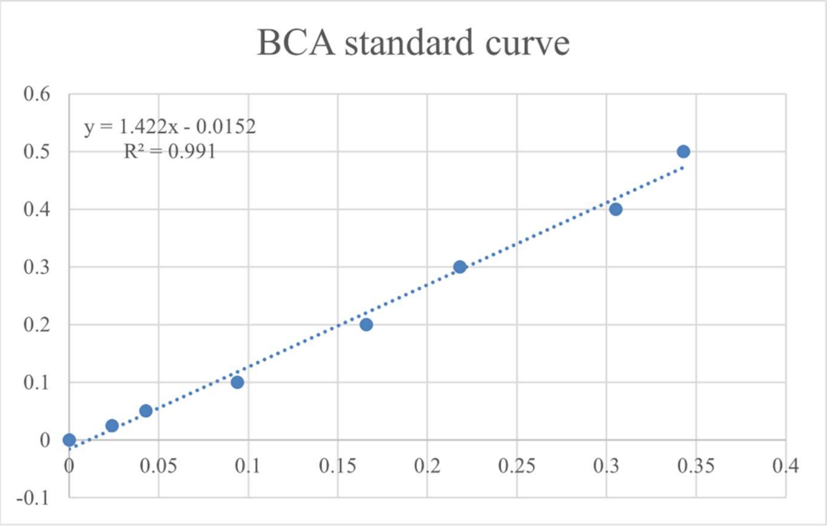

NO.	1	2	3	4	5	6	7	8
**STD（A562）**	0	0.024	0.043	0.094	0.166	0.218	0.305	0.343
**CONC（mg/ml）**	0	0.025	0.05	0.1	0.2	0.3	0.4	0.5
**Sample（A562）**	0	0.008	0.008	0.03	0.031	0.074	0.089	0.131
**CONC（mg/ml）**	0	0	0	0.027	0.028	0.09	0.111	0.171

Figure S3. (a) BCA method to determine the amount of magnetic nanoparticle-conjugated antibodies.

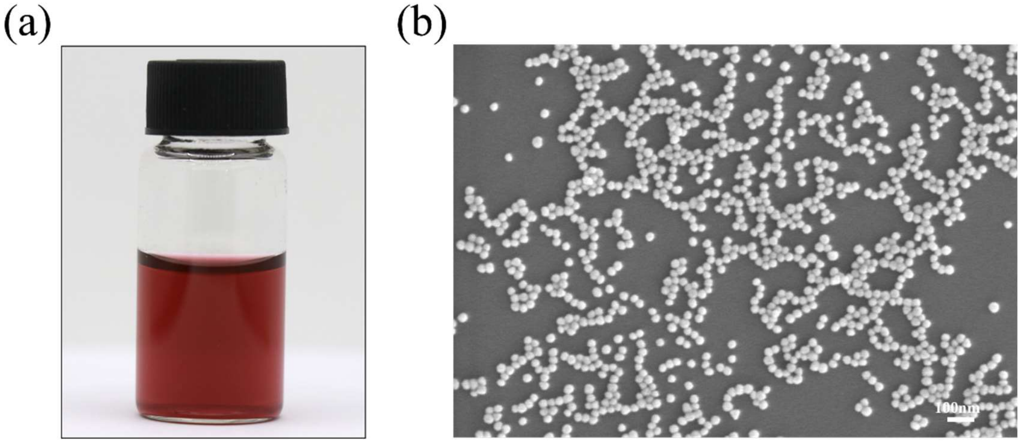

Figure S4. (a) Photograph of gold nanoparticles; (b) scanning electron microscope image of gold nanoparticles.

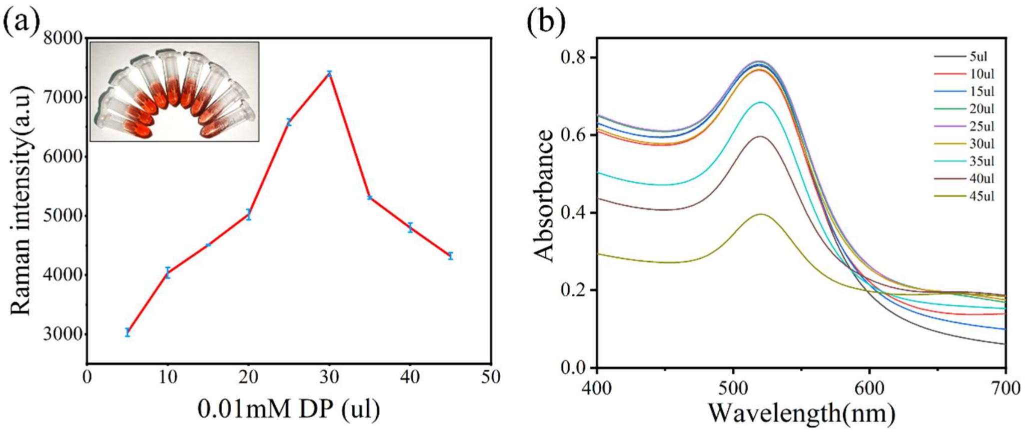

Figure S5. (a) The volume of bipyridine addition in relation to Raman strength; (b) the volume of DP added corresponds to the UV absorption spectrum.

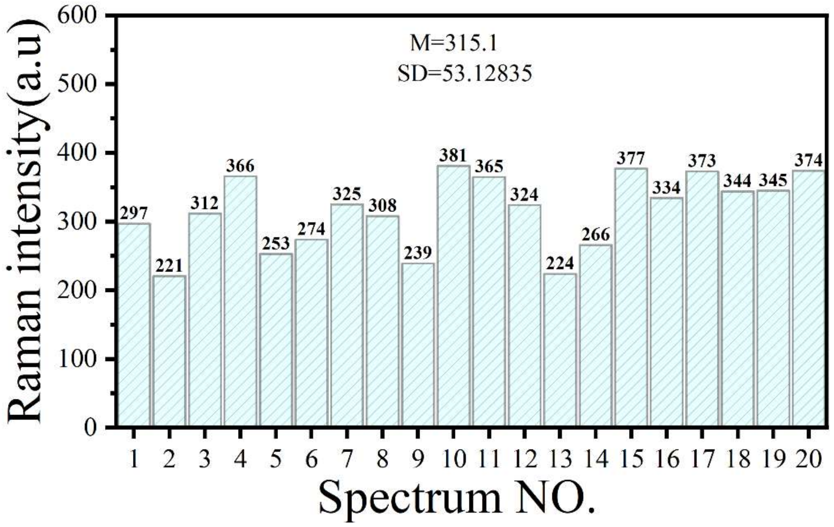

Figure S6. Raman signal value for 20 blank detections at 1612cm $^{-1}$.
